# Mortality rate and predictors of mortality of pediatric sepsis and septic shock in a Moroccan tertiary care center

**DOI:** 10.3389/fped.2026.1672491

**Published:** 2026-08-28

**Authors:** Ouissal Aissaoui, Ibtissam Nabih, Youssef Idbaha, Abdelaziz Chlilek, Ahmed Aziz Bousfiha

**Affiliations:** 1 Pediatric Anesthesiology and Intensive Care Unit, Abderrahim Harouchi Mother-Child hospital, Laboratory of Clinical Immunology, Infection and Inflammation (LICIA), Faculty of Medicine and Pharmacy of Casablanca, Hassan II University of Casablanca, Casablanca, Morocco; 2 Pediatric Infectious Diseases and Clinical Immunology Unit, Department of Pediatrics, Abderrahim Harouchi Mother-Child hospital, Laboratory of Clinical Immunology, Infection and Inflammation (LICIA), Faculty of Medicine and Pharmacy of Casablanca, Hassan II University of Casablanca, Casablanca, Morocco

**Keywords:** sepsis, septic shock, mortality, children, PICU, pSOFA, logistic regression

## Abstract

**Background:**

Sepsis is the leading cause of child mortality worldwide. Pediatric sepsis-related mortality in Africa is estimated to be as high as 40%. We aimed to assess sepsis-related mortality and to identify predictors of mortality in pediatric sepsis.

**Methods:**

We conducted a retrospective observational study of children admitted to the PICU for sepsis or septic shock. Statistical analysis was performed using Jamovi version 2.3.28. Continuous variables are expressed as mean ± standard deviation (SD) and categorical variables as frequencies and percentages. The Mann–Whitney *U* test assessed the association between the pediatric sequential sepsis-related organ failure assessment “pSOFA” score and mortality. Logistic regression was used to identify mortality predictors. Model performance was assessed based on discrimination and calibration. Statistical significance was set at *p* < 0.05.

**Results:**

We enrolled 130 patients. The mean age was 32.5 months (SD: 47.12, range: 1–180). The identified infection sites were pneumonia (53.1%), central nervous system (23.8%), gastrointestinal tract (15.4%), bloodstream (4.5%), bone and joint (2.3%), and urinary tract (0.8%). Biological assessment showed the following mean values: 14,076 E/mm^3^ for leukocytes (SD: 9,162, range: 350–59,100), 349 x 10^3^ E/mm^3^ for platelets (SD: 214, range: 10–939) and 111 mg/L for C-reactive protein “CRP” (SD 122, range:1–480). The median pSOFA score was six (range: 2–18). A microorganism was isolated in 32.3% of cases, including SARS-CoV-2 (5.2%), Streptococcus pneumoniae (3.7%), respiratory syncytial virus (3%), and Pseudomonas aeruginosa (2.3%). The overall mortality rate was 50%. Statistical analysis showed an association between the pSOFA score and mortality (*p* < 0.001). Lower platelet count, vasopressor use, and mechanical ventilation were independently associated with mortality. The logistic regression model performed well with a *p*-value < 0.001 and a McFadden R^2^ of 0.415. The area under the ROC curve was 0.888. The sensitivity, specificity, and accuracy were 88.9%, 85.1%, and 87.0%, respectively, with acceptable calibration.

**Conclusion:**

Our research assessed pediatric sepsis in Morocco, a lower-middle-income North African country. Mortality rates were higher compared to high-income countries. The pSOFA score was effective in predicting severity and mortality. Lower platelet count, vasopressor use, and mechanical ventilation were identified as independent predictors of mortality.

## 1 Introduction

Sepsis is recognized as a public health priority. The latest global report on the epidemiology and burden of sepsis, published by the World Health Organization (WHO), revealed 48.9 million incident cases and 11 million deaths, representing nearly one-fifth of all global mortality. The pediatric population is severely impacted, as approximately 20 million cases, which represent over 40% of the global total, occur in children under five**.** Pediatric sepsis is the leading cause of pediatric mortality worldwide. According to the same report, sepsis was responsible for approximately 2.9 million deaths in children under five, accounting for 26.4% of all sepsis-related mortality worldwide. The Sepsis Prevalence, Outcomes and Therapies study (SPROUT) published in 2015 estimated a pediatric intensive care (PICU) mortality rate of 25% with considerable disparity between continents. The following rates were reported in the study: 21% in North America, 29% in Europe, 32% in Australia/New Zealand, 40% in Asia, 11% in South America, and 40% in Africa (1). A recently published systematic review and meta-analysis reported a global pediatric sepsis case fatality rate of 25%, with pooled case fatality rates of 19.7% in developed countries and 31.9% in developing countries (2). A recent study assessing the epidemiology of sepsis in 144 PICUs in Brazil, an upper-middle-income country, revealed a mortality rate of 19.8% (3), whereas studies from Pakistan, a lower-middle-income country, revealed mortality rates exceeding 52% (4). Additionally, the burden of the disease extends beyond mortality, with survivors at risk of short-term complications and longer-term functional, neurocognitive, and psychological impairment.

Focusing on North Africa, the heterogeneity of sepsis definitions and inclusion criteria, as well as the characteristics of the healthcare system, form considerable barriers to generalization. For contextualization, we report herein published data originating from neighboring countries to Morocco. In Egypt, a large prospective cohort from three tertiary PICUs in Cairo used Sepsis-3 derived definitions and reported a mortality rate of 26.4%, with higher mortality in hospital-acquired sepsis than community-acquired sepsis (5). Conversely, a prospective cohort study from Alexandria, including children aged from 1 month to 13 years and using SIRS-based pediatric consensus definitions, reported a 28-day mortality of 35.3% (6). In Tunisia, a retrospective septic shock cohort reported a 45.7% mortality rate (7). Several factors contribute to sepsis mortality in low and middle income settings, including difficult access to healthcare and low vaccination coverage, malnutrition, delayed referral and access to the pediatric intensive care unit (PICU) due to insufficient beds, delayed recognition, shortage of staff, antimicrobial resistance, lack of standardized written local protocols, higher burden of pediatric communicable infectious diseases, and unavailability of advanced monitoring and organ support (8, 9).

The conceptualization of pediatric sepsis has undergone a significant paradigm shift, evolving from the initial Systemic Inflammatory Response Syndrome (SIRS)-based framework of 1991 to the contemporary understanding of a dysregulated host immune response leading to life-threatening organ dysfunction. While early definitions like the 2005 International Pediatric Sepsis Consensus Conference (IPSCC) relied heavily on inflammatory markers, the current Phoenix Sepsis Score aligns with the adult Sepsis-3 criteria by focusing on acute organ failure and abandoning the term “severe sepsis”. According to the 2024 international consensus criteria for pediatric sepsis and septic shock (10), sepsis is identified as an organ dysfunction secondary to a dysregulated immune response to an infection and is operationalized by the Phoenix Sepsis Score. This definition replaces the old paradigm linking sepsis severity to the microorganism's virulence with one that emphasizes the pivotal role of the host's immune system in severity and outcome. In the same context, recent literature identifies the role of underlying inborn errors of immunity (IEI) in the pathophysiology of pediatric sepsis. For instance, the prevalence of IEI in children with unknown immune deficiencies has been reported to range from 20 to 61% according to a systematic review (11). The two main studies on this topic used whole-exome sequencing (WES). The first study was the study by Borghesi et al., where they identified a prevalence of pathogenic variants of IEI of 20% in a cohort of 176 children with blood culture-positive sepsis (12). The second study, by Kernan et al., reported a prevalence of 44% in a cohort of 330 previously healthy children admitted to the PICU for severe sepsis (13). In our setting, we previously reported a prevalence of IEI of 17% using whole exome sequencing in a cohort of children admitted to our PICU for sepsis (14).

The World Health Organization (WHO) has recognized sepsis as a priority health problem, and stated that sepsis control programs should be adapted to the unique challenges encountered in low- and middle-income settings (15). Nevertheless, the paucity of pediatric sepsis data remains a barrier to quality and outcome improvement strategies (16, 17). Data on pediatric sepsis and septic shock in Morocco, a North-African lower-middle-income country, have never been published previously, and filling this literature gap constitutes a major first step towards developing programs aimed at reducing sepsis-related mortality and morbidity in our setting.

We aimed to assess sepsis and septic shock-related mortality in children admitted to a tertiary care PICU in Morocco as a primary objective, and to identify predictors of mortality as a secondary aim through a retrospective observational study tailored to the unique characteristics of our population and to the pronounced challenges of our healthcare system.

## 2 Methods

### 2.1 Study design and place

We conducted a retrospective observational cohort study including children admitted to our PICU with community-acquired sepsis and/or septic shock. The study was reported in accordance with the Strengthening the Reporting of Observational Studies in Epidemiology (STROBE) guidelines for observational studies (18). It was conducted between April 2021 and December 2023, a period that partially overlapped with the COVID-19 pandemic. It took place in the Pediatric Intensive Care Unit of Abderrahim Harouchi Mother-Child Hospital, a public tertiary-care referral and academic center affiliated with the Ibn Rochd University Hospital. The hospital provides specialized medical and surgical care and receives patients from Casablanca and the surrounding regions, covering a catchment population of 7.7 million inhabitants (19). Children aged 0 to 18 years old account for 31.6% of the population, including approximately 2.4 million individuals. Within the regional healthcare network, the Mother-Child hospital serves as a referral center for critically ill pediatric patients requiring advanced monitoring and organ support and is equipped with approximately 420 pediatric beds and one multidisciplinary PICU. The PICU has 12 beds and admits medical and surgical pediatric and neonatal cases, and operates at approximately 110% of its capacity throughout the year. The medical staff consists of four senior consultants, six residents, and three interns. The Nursing staff operates according to a patient-to-nurse ratio of 3:1.

### 2.2 Definitions

Sepsis was defined as a bacterial, viral, or fungal infection associated with organ dysfunction. Organ dysfunction was assessed using the age-adapted sequential sepsis-related organ failure assessment score, also known as pediatric SOFA (pSOFA). A pSOFA score ≥ 2 was considered significant. This score, as shown in the appendix, evaluates the following six organ dysfunctions: respiratory, neurologic, cardiovascular, hematological, hepatic, and renal, and was calculated during the first 24 h of admission. Septic shock was defined as sepsis with a cardiovascular dysfunction persisting after an initial fluid resuscitation of 40 mL/kg of crystalloids and motivating the use of vasopressors. Sepsis and septic shock status were classified according to the admission presentation and did not account for progression during the first 24 h after PICU admission.

### 2.3 Inclusion and exclusion criteria

We retrospectively screened all medical records from April 2021 to December 2023.

**Figure 1:**
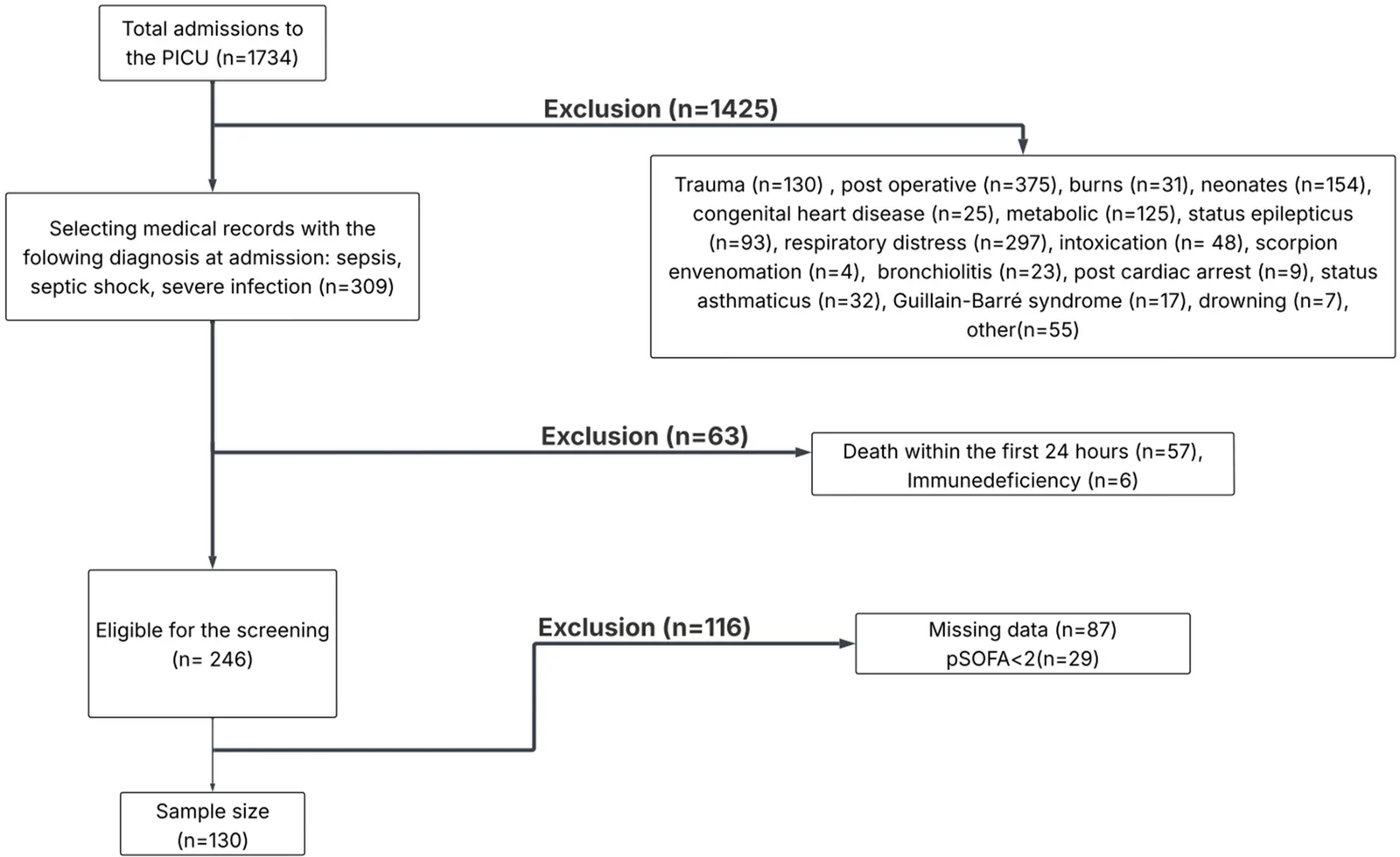
Flow chart of the study population and patient selection process. PICU: pediatric intensive care unit; pSOFA: pediatric sequential sepsis-related organ failure assessment.

We selected charts with admission diagnoses of sepsis, septic shock, or severe infection, including pneumonia, meningitis, encephalitis, urinary tract infection, bone and joint infection, and gastroenteritis, and retained only children who met the study definition of sepsis. The following admission conditions were excluded: trauma, postoperative care, burns, neonatal conditions, metabolic decompensation, status epilepticus, respiratory distress, intoxication, scorpion envenomation, bronchiolitis, post-cardiac arrest, status asthmaticus, Guillain-Barré syndrome, drowning, congenital heart disease, and other accidental conditions. Neonates, defined as infants younger than 28 days of age, were excluded from the study. Children with known primary or secondary immunodeficiency were excluded from the study, as were patients who died within the first 24 h of admission. We secondarily excluded patients with missing data and those with a pSOFA score < 2. The exclusion criteria are presented in the study flowchart (Figure 1).

### 2.4 Data collection

Demographic and clinical data collected included age, medical history, presenting symptoms and vital signs at admission. The biological tests included complete blood counts, C-reactive protein (CRP) levels, and liver and kidney function tests, which were performed during the first 24 h of admission. Microbiological assessment included blood, sputum, urine, and cerebrospinal fluid cultures as well as multiplex PCR syndromic testing on nasopharyngeal swabs (when available), which were performed according to the suspected infection site. Data were collected using a Microsoft Excel spreadsheet.

### 2.5 Statistical analysis

Descriptive statistics were calculated and expressed using means, medians, standard deviations (SD), and minimum and maximum values for continuous variables. Frequencies and percentages were used to describe categorical variables. The Chi-square test was used to assess associations between categorical variables. *T*-tests were used to compare continuous and categorical variables in univariate analyses. The normality of distribution was checked using the Shapiro–Wilk test and homogeneity of variance using Levene's test. In case of violation of the assumption of normality and homogeneity, we used the Mann–Whitney *U* test. Therefore, to assess the association between the pSOFA score and mortality, we used the Mann–Whitney *U* test. Binomial logistic regression analysis was used to identify independent predictors of mortality. Statistical significance was set at *p* < 0.05. The collinearity between variables was assessed using the variance inflation factor (VIF), with a VIF > 5 considered suggestive of problematic collinearity. We aimed to include 7 variables in the logistic regression model. We used the rule of thumb of 10 events per variable with an estimated mortality rate of 55% (according to previous studies from the same unit). The calculation yielded a sample size of 128 patients. We included 130 patients with retrospective consecutive sampling. The model fit was assessed using McFadden's R^2^. The following predictive parameters were evaluated: accuracy, sensitivity, and specificity as well as the area under the ROC curve. Calibration of the logistic regression model was assessed by comparing the mean predicted probability with the observed mortality rate in the cohort and by calculating the Brier score. Statistical analysis was performed using JAMOVI version 2.3.28.

### 2.6 Ethical considerations

The study complied with the regulations and guidelines applicable to our institutional settings and ethical approval was obtained from the Ibn Rochd University Hospital institutional ethical board.

## 3 Results

During the study period, we recorded 1,734 admissions. Initially, 309 met the inclusion criteria for sepsis, septic shock, and severe infection. After exclusion of patients with death within the first 24 h, missing data, pSOFA < 2, and immunodeficiencies, the final study population consisted of 130 patients.

### 3.1 General characteristics

A total of 130 children were included. The mean age at admission was 32.5 ± 47.12 months. The mean length of ICU stay was seven days. The additional patient characteristics are shown in Table 1.

**Table 1:** General characteristics of the study population.

Variable	Mean	Median	SD	Min	Max	Counts	% of total
Age (months)	32.51	9.00	47.12	1	180	—	—
<2 years old	—	—	—	—	—	91	70
2–5 years old	—	—	—	—	—	12	9.2
>5 years old	—	—	—	—	—	27	20.8
Gender							
Males	—	—	—	—	—	77	59.2
Females	—	—	—	—	—	53	40.8
Comorbidities						15	11.5
Perinatal asphyxia	—	—	—	—	—	5	—
Convulsions	—	—	—	—	—	3	—
Hydrocephalus	—	—	—	—	—	1	—
Wheezing	—	—	—	—	—	4	—
Diabetes	—	—	—	—	—	2	—
Biological markers							
Leukocytes (E/mm^3^)	14,076.55	13,060	9,162.77	350	59,100	—	—
Neutrophils (E/mm^3^)	8,792.77	6,560	7,696.65	0	50,180	—	—
Lymphocytes (E/mm^3^)	3,658.23	2,720	2,644.19	150	16,350	—	—
Platelets (x10^3^ E/mm^3^)	349	368	215	10	939	—	—
CRP	111.21	68	122	1	480	—	—
Vasopressors	—	—	—	—	—	63	48.5
Mechanical ventilation	—	—	—	—	—	53	40.8
Length of stay	7.34	5.0	8.78	1	60	—	—
pSOFA	6.72	6.0	3.12	2	18	—	—
Mortality	—	—	—	—	—	65	50

Data are presented as mean, median, standard deviation (SD), minimum (Min), and maximum (Max) for continuous variables and as counts and percentages for categorical variables.

SD, standard deviation; Min, minimum; Max, maximum; CRP, C-reactive protein; pSOFA, pediatric sequential sepsis-related organ failure assessment.

### 3.2 Infection site and microorganism identification

Pneumonia was the leading cause of sepsis in our cohort and was present in 53.1%. Central nervous system (CNS) infection, including meningitis and meningoencephalitis, was identified in 23.8% followed by gastrointestinal tract infection in 15.4%, bloodstream infection in 4.5%, bone and joint infection in 2.3%, and urinary tract infection in 0.8% of the cases.

Microorganisms were identified in 32.3% of cases, with bacterial sepsis in 18.5%, viral sepsis in 12.3%, and fungal sepsis in 1.5% of cases. The results are presented in Table 2.

**Table 2:** Microbiological findings.

Microorganism	*n*	%
Bacterial	24	18.5
Bacteroides spp	1	0.7
Mycobacterium tuberculosis	2	1.5
Haemophilus influenzae b	2	1.5
E. coli	2	1.5
Klebsiella pneumoniae	2	1.5
Neisseria meningitidis	2	1.5
Pseudomonas aeruginosa	3	2.3
Streptococcus pneumoniae	5	3.7
Porphyromonas asaccharolytica	1	0.7
Ralstonia pickettii	2	1.5
Staphylococcus aureus	2	1.5
Viral	16	12.3
Parainfluenza virus	1	0.7
HSV	2	1.5
Influenza A virus	2	1.5
SARS-CoV-2	7	5.2
RSV	4	3
Fungal	2	1.5
Candida albicans	1	0.7
Candida parapsilosis	1	0.7
No micro-organism identified	88	68

HSV, herpes simplex virus; SARS-CoV-2, severe acute respiratory syndrome coronavirus 2; RSV, respiratory syncytial virus; *n*, counts; %, percentages.

### 3.3 Sepsis management

In our cohort, all children received fluid boluses at admission and broad-spectrum antibiotics in accordance with local protocols previously described (20). Sixteen per cent of patients received antivirals. In our setting, antiviral therapy was mainly administered in cases of meningitis or meningoencephalitis as empiric coverage for possible herpes simplex virus infection. During the first 24 h of admission, mechanical ventilation was used in 40.8% of the cases and vasopressors in 48.5% of cases. Renal replacement therapy was performed in 2.3%.

### 3.4 Severity and mortality predictors

Sepsis was identified in 95 cases (73.1% of total) and septic shock in 35 cases (26.9% of total). Sepsis-related mortality was 42% (40 of 95 patients died) and septic shock-related mortality was 71% (25 of 35 patients died). The overall mortality rate was 50% (*n* = 65).

**Figure 2:**
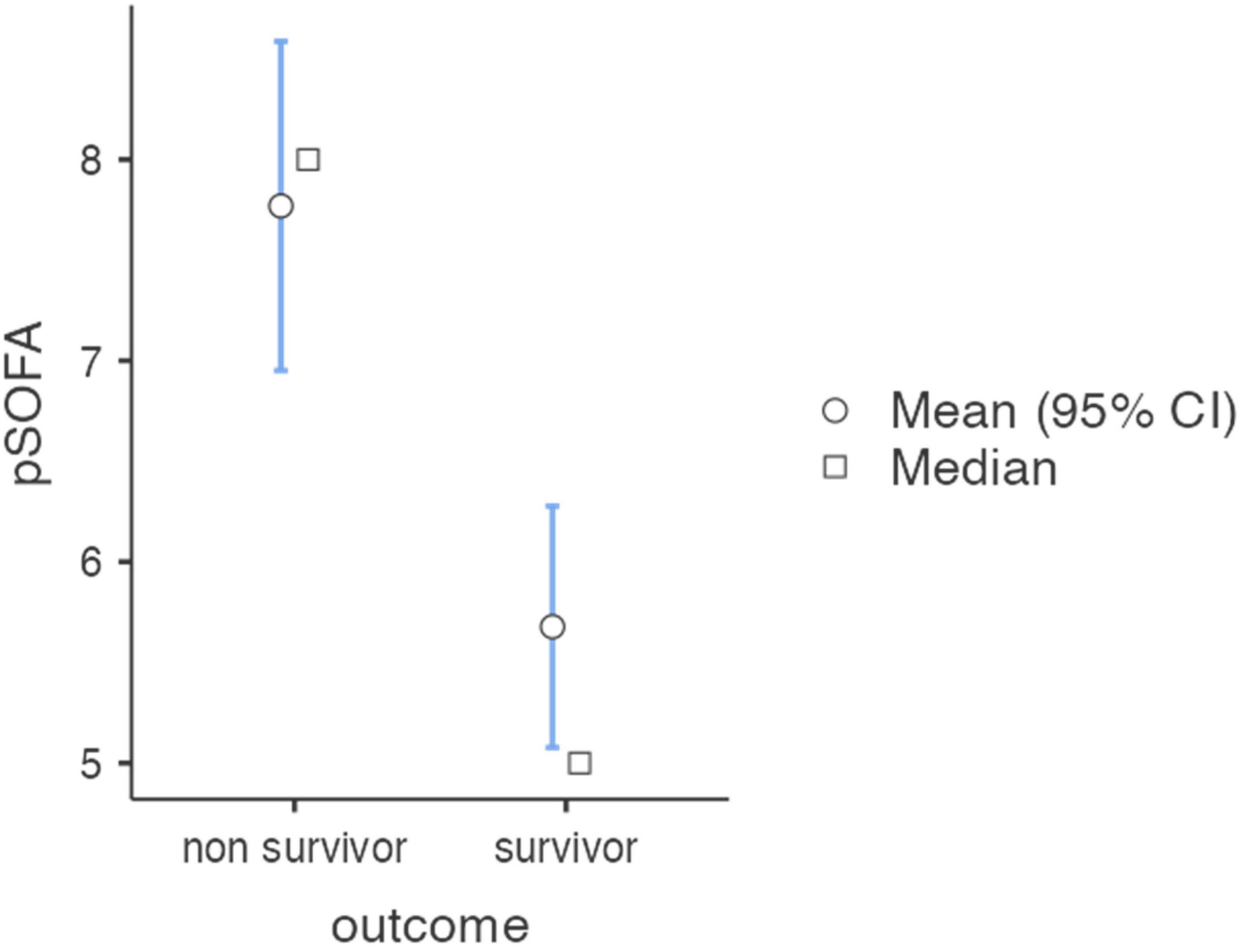
Comparison of pSOFA scores between survivors and non-survivors. The pSOFA score was significantly higher among non-survivors (Mann–Whitney *U* test, *p* < 0.001). pSOFA, pediatric sequential sepsis-related organ failure assessment score; CI, confidence interval.

Vasopressor use was more frequent than the proportion of patients classified as having septic shock at admission, likely reflecting clinical deterioration during the first 24 h, whereas septic shock status was assigned according to the admission presentation. Multi-organ failure was assessed using the pSOFA score. The pSOFA score was higher among the non-survivors, as shown in Figure 2. Using the Mann–Whitney *U* test, we found an association between pSOFA score and mortality (*p* < 0.001). We evaluated variables independently associated with mortality using logistic regression analysis. We found no significant association between mortality and the following variables: age, neutrophils, lymphocytes, or CRP level. We identified a statistically significant association between lower platelet count, mechanical ventilation, vasopressor use, and mortality (Table 3).

**Table 3:** Binomial logistic regression analysis of predictors of mortality.

Predictor	Estimate	SE	*Z*	*p*	Odds ratio	95% CI	
						Lower	Upper
Intercept	−0.13316	1.02313	−0.130	0.896	0.875	0.118	6.502
mechanical ventilation:							
yes—no	1.75499	0.63973	2.743	0.006	5.783	1.651	20.264
vasopressors:							
yes—no	2.63300	0.62199	4.233	<.001	13.915	4.112	47.091
CRP (mg/L)	−3.98 × 10^−4^	0.00251	−0.159	0.874	1.000	0.995	1.005
age	−0.00490	0.00804	−0.609	0.543	0.995	0.980	1.011
neutrophils (E/mm3)	−9.46 × 10^−6^	4.42 × 10^−5^	−0.214	0.830	1.000	1.000	1.000
lymphocytes (E/mm3)	−5.17 × 10^−5^	1.28 × 10^−4^	−0.403	0.687	1.000	1.000	1.000
platelets (x10³/mm3)	−0.00349	0.00149	−2.347	0.019	0.997	0.994	0.999

CI, confidence interval; SE, standard error; *Z*, *z*-statistic; *p*, *p*-value; CRP, C-reactive protein. Odds ratios are presented with 95% confidence intervals. Estimates represent the log odds of non-survival vs. survival. Vasopressor use, mechanical ventilation, and lower platelet count showed statistically significant independent associations with mortality.

### 3.5 Model performance

**Figure 3:**
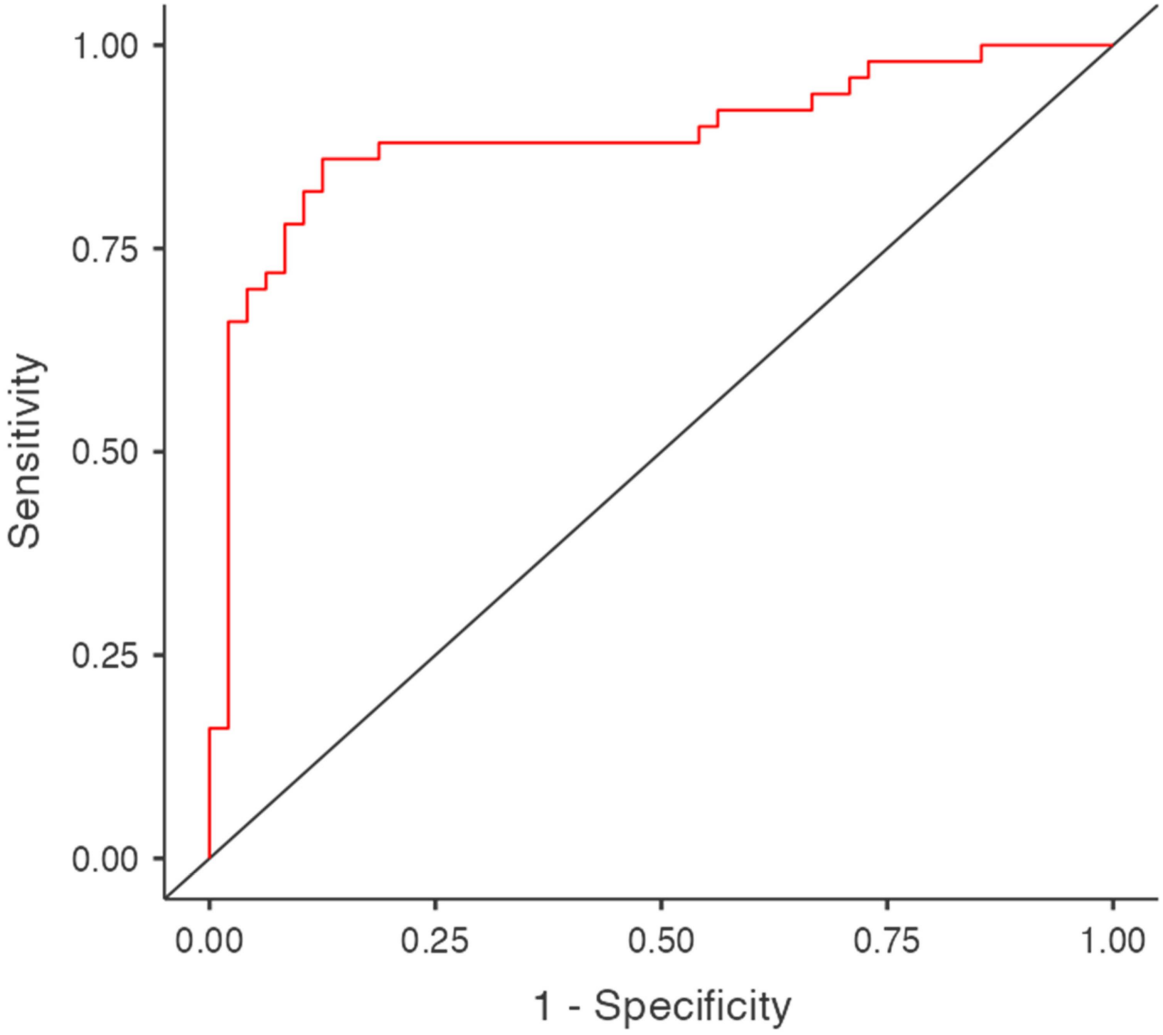
The receiver operating characteristic (ROC) curve of the logistic regression model. The model demonstrated good discrimination, with an AUC of 0.888. At a probability threshold of 0.5, sensitivity was 88.9%, specificity was 85.1%, and overall accuracy was 87.0%.

The logistic regression model showed a McFadden pseudo-R^2^ of 0.415, with an overall model chi-square of 52.9 (df = 7, *p* < 0.001). The model demonstrated good discrimination, with an AUC of 0.888 (Figure 3). At a probability threshold of 0.5, sensitivity was 88.9%, specificity was 85.1%, and overall accuracy was 87.0% (Table 4). Calibration of the logistic regression model appeared acceptable. The mean predicted probability of mortality was 0.51, which was close to the observed mortality rate of 50% in the cohort. The Brier score was 0.119, suggesting satisfactory overall predictive performance.

**Table 4:** Model fit and predictive performance measures of the logistic regression model.

Model	R^2^_McF_	Overall model test		
		*χ* ^2^	df	*p*
1	0.415	52.9	7	<.001

Accuracy	Specificity	Sensitivity	AUC
0.870	0.851	0.889	0.888

R^2^_McF_, McFadden pseudo-R^2^; *χ*^2^, chi-square statistic; df, degrees of freedom; *p*, *p*-value; AUC, area under the receiver operating characteristic curve. The model demonstrated good discrimination, with an AUC of 0.888. At a probability threshold of 0.5, sensitivity was 88.9%, specificity was 85.1%, and overall accuracy was 87.0%.

## 4 Discussion

Our study provides data on pediatric sepsis and septic shock in a Moroccan tertiary PICU, a setting for which published evidence remains scarce. The cohort enrolled included 130 patients with a mean age of 32.5 months. The main site of infection was pneumonia, followed by infections of the central nervous system and gastrointestinal tract. A microorganism was isolated in 32.3% of cases. The median pSOFA score was six, with significantly higher scores in non-survivors. The overall mortality rate was 50%. Statistical analysis showed an association between the pSOFA score and mortality (*p* < 0.001). Lower platelet count, vasopressor use, and mechanical ventilation were independently associated with mortality. The logistic regression model performed well with a *p*-value < 0.001 and a McFadden R^2^ of 0.415. The area under the ROC curve was 0.888. The sensitivity, specificity, and accuracy were 88.9%, 85.1%, and 87.0%, respectively, with acceptable calibration.

Sepsis is defined as a dysregulated immune response to infection that leads to multi-organ failure (21). Septic shock is a subset of sepsis, in which profound circulatory, cellular, and metabolic abnormalities are associated with a greater risk of mortality. Several scores were adapted to assess sepsis related organ failure and to predict mortality including “pSOFA” score, the Pediatric Logistic Organ Dysfunction-2 “PELOD-2” score, the 2005 International Pediatric Sepsis Consensus Conference “IPSCC” score, the Pediatric Organ Dysfunction Information Update Mandate (PODIUM) score and most recently the Phoenix Sepsis Score, following the 2024 international consensus criteria for pediatric sepsis and septic shock. These scores showed comparable sensitivity in predicting culture-positive sepsis-related mortality in pediatric intensive care units (22). However, they have rarely been assessed in Morocco, where their performance remains unclear. In our study, the pSOFA score was significantly higher in non-survivors than in survivors, suggesting that a greater burden of organ dysfunction at admission is associated with a higher risk of death. This finding is in line with previous studies showing that organ dysfunction scores are useful for severity assessment and prognostic stratification in pediatric sepsis. A literature review compiling 14 studies and including 70,194 participants reported a pooled sensitivity for age-adjusted SOFA of 0.82 (95% CI: 0.74–0.88), a pooled specificity of 0.62 (95% CI: 0.45–0.77) and an area under the summary receiver operating characteristic curve of 0.82 (95% CI: 0.79–0.85), concluding that the pSOFA score is a powerful tool for predicting sepsis-related mortality in the PICU (23). Our study supports this finding, with a strong association between pSOFA score and mortality (*p*-value < 0.001).

The mortality observed in our cohort was substantially higher than that reported in many studies from high-income settings. It was also higher than the overall mortality reported in large international cohorts. However, these findings should be interpreted in the context of a resource-limited African setting. The study was conducted in a tertiary university hospital in Casablanca, covering a catchment population of 7.7 million inhabitants. This hospital receives a large number of referrals, including severe and advanced cases from a broad catchment area of 19,448 km^2^. In such contexts, several factors may contribute to this excess mortality, including delayed presentation, delayed referral to intensive care, limited PICU capacity, restricted access to advanced organ support, and the high severity of illness at admission related to the tendency for only the most critically ill children to be admitted to ICU because of restricted bed availability. Furthermore, the remarkably low rate of renal replacement therapy (2.3%) observed in our high-mortality cohort likely reflects constrained access to specialized equipment and bedside resources rather than a lack of clinical necessity among these critically ill patients. Additionally, our unit functions under sustained overcrowding, which may further affect timely escalation of care and resource availability. Social and cultural factors may also influence healthcare-seeking behavior and contribute to delayed access to care. These population and health-system dynamics are important for understanding the high mortality observed in our cohort and should be considered when comparing our findings with those from better-resourced settings.

The microorganism identification rate was low in our study, since bacterial, viral, and fungal infections were documented in only 32.3% of cases. Blood culture positivity was low, most likely due to the common use of antibiotics before the PICU admission. Additionally, the study period overlapped with the COVID-19 pandemic and post-pandemic era, which were characterized by the predominance of viral infections that were not identified in the absence of extensive viral diagnostic panels. Nevertheless, previously published literature stated that culture-positive and culture-negative sepsis present with similar characteristics and outcomes in adult and pediatric populations (24, 25).

In multivariable analysis, lower platelet count was independently associated with mortality. This finding is clinically plausible, as thrombocytopenia or lower platelet values may reflect sepsis-related coagulopathy, platelet consumption, endothelial injury, and overall disease severity.

Thrombocytopenia has been associated with an increased length of stay, prolonged need for organ support, major bleeding effects, and mortality in adult patients with septic shock (26). A recent retrospective cohort study conducted on pediatric septic shock patients compared two subgroups, one with thrombocytopenia and the other without thrombocytopenia, and demonstrated that thrombocytopenia during the first 24 h of septic shock onset was predictive of increased 28-day mortality (27). In our study, platelet count was entered into the model as a continuous variable; therefore, the results should be interpreted as an association between lower platelet values and death rather than as a categorical thrombocytopenia effect.

Vasopressor use and mechanical ventilation were also strongly associated with mortality. These variables likely reflect advanced organ dysfunction and greater illness severity rather than isolated causal determinants of death. Children requiring vasopressor support have severe circulatory failure, whereas those requiring invasive ventilation generally have marked respiratory and/or neurologic compromise. Their strong association with mortality in our model, therefore, reinforces the central role of multiorgan dysfunction in sepsis outcome.

### 4.1 Limitations

Our study has some limitations. It was a retrospective study based on reviewing patient charts and biological data. Important sepsis markers such as lactate and procalcitonin are lacking. In fact, many sepsis biomarkers are unavailable in low-resource public hospitals owing to their cost. In this context, the clinical expertise of the medical team, and most crucially of senior clinicians, plays a central role in the early recognition of clinical deterioration and in guiding sepsis management, making bedside clinical judgment a cornerstone of sepsis care in these settings. Microorganism identification is not always possible because of the non-standardization of molecular diagnosis using PCR, and blood cultures are rarely positive because of prior antibiotic administration and the prevalence of viral sepsis during the COVID-19 pandemic and post-pandemic period.

Furthermore, the study population included patients with sepsis and septic shock, which explains the heterogeneity of the results, especially regarding mortality rates between the two previously mentioned subgroups. On the other hand, the diagnosis of sepsis or septic shock was assigned on admission to the PICU and did not consider the evolutionary aspect from sepsis to septic shock during the first 24 h, explaining the disparity between the use of vasopressors and the proportion of septic shock patients.

Additionally, our study was conducted before the publication of the new pediatric sepsis definition using the Phoenix Sepsis Score; therefore, the sepsis definition was extrapolated from Sepsis-3, and the pSOFA score was used as a validated severity score (28). More prospective data should be obtained using the new definition to assess the pediatric sepsis burden in the future.

## 5 Conclusion

This study aimed to assess pediatric sepsis in the region of Casablanca, Morocco, and thus relate data from a tertiary care, resource-limited, North-African setting. Mortality was high, likely reflecting the combined effect of delayed presentation, referral of the most severe cases, limited PICU capacity, restricted access to advanced diagnostics and organ support, and broader structural constraints affecting care delivery. Therefore, mortality rates should not be directly compared with those reported from high-resource settings. Within our context, the pSOFA score was effective in predicting severity and mortality and appeared to be a useful clinical tool for severity assessment and prognostic stratification. Lower platelet count, vasopressor use, and mechanical ventilation were independently associated with mortality, reflecting the impact of organ dysfunction on pediatric sepsis outcome.

## Data Availability

The raw data supporting the conclusions of this article will be made available by the authors, without undue reservation.

## Appendix: The pSOFA score.

**Table:**

	0	1	2	3	4
Respiratory					
PaO2/FiO2Or	≥400	300–399	200–299	100–199*	100*
SpO2/FiO2	≥292	264–291	221–264	148–220*	<148*
Coagulation					
Platelet count	≥150	100–149	50–99	20–49	<20
Hepatic					
Bilirubin, mg/dL	<1.2	1.2–1.9	2.0–5.9	6.0–11.9	>12.0
Cardiovascular					
Mean arterial pressure by age group (mmHg) or vasoactive infusion (mcg/kg/min)					
<1 month	≥46	<46	Dopamine ≤ 5 or Dobutamine	Dopamine > 5 or epinephrine ≤ 0.1 or norepinephrine ≤ 0.1	Dopamine > 15 or epinephrine > 0.1 or norepinephrine > 0.1
1–11 months	≥55	<55			
12–23 months	≥60	<60			
24–59 months	≥62	<62			
60–143 months	≥65	<65			
144–216 months	≥67	<67			
>216 months	≥70	<70			
Neurologic					
GCS	15	13–14	10–12	6–9	<6
Renal					
Creatinine by age group, mg/dL					
<1 months	<0.8	0.8–0.9	1.0–1.1	1.2–1.5	≥1.6
1–11 months	<0.3	0.3–0.4	0.5–0.7	0.8–1.1	≥1.2
12–23 months	<0.4	0.4–0.5	0.6–1.0	1.1–1.4	≥1.5
24–59 months	<0.6	0.6–0.8	0.9–1.5	1.6–2.2	≥2.3
60–143 months	<0.7	0.7–1.0	1.1–1.7	1.8–2.5	≥2.6
144–216 months	<1.0	1.0–1.6	1.7–2.8	2.9–4.1	≥4.2
>216 months	<1.2	1.2–1.9	2.0–3.4	3.5–4.9	≥5

PaO2: partial pressure of oxygen, FiO2: fraction of inspired oxygen, GCS: Glasgow coma scale.

* with respiratory support.
